# The maintenance of sex: Ronald Fisher meets the Red Queen

**DOI:** 10.1186/1471-2148-13-174

**Published:** 2013-08-21

**Authors:** David Green, Chris Mason

**Affiliations:** 1 Department of Anatomy, University of Otago Medical School, Great King Street, Dunedin 9016, New Zealand

**Keywords:** Maintenance of sex, Advantageous mutation, Frequency-dependent selection, Red Queen, Computational model

## Abstract

**Background:**

Sex in higher diploids carries a two-fold cost of males that should reduce its fitness relative to cloning, and result in its extinction. Instead, sex is widespread and clonal species face early obsolescence. One possible reason is that sex is an adaptation that allows organisms to respond more effectively to endless changes in their environment. The purpose of this study was to model mutation and selection in a diploid organism in an evolving environment and ascertain their support for sex.

**Results:**

We used a computational approach to model finite populations where a haploid environment subjects a diploid host to endlessly evolving change. Evolution in both populations is primarily through adoption of novel advantageous mutations within a large allele space. Sex outcompetes cloning by two complementary mechanisms. First, sexual diploids adopt advantageous homozygous mutations more rapidly than clonal ones under conditions of lag load (the gap between the actual adaptation of the diploid population and its theoretical optimum). This rate advantage can offset the higher fecundity of cloning. Second, a relative advantage to sex emerges where populations are significantly polymorphic, because clonal polymorphism runs the risk of clonal interference caused by selection on numerous lines of similar adaptation. This interference extends allele lifetime and reduces the rate of adaptation. Sex abolishes the interference, making selection faster and elevating population fitness. Differences in adaptation between sexual and clonal populations increase markedly with the number of loci under selection, the rate of mutation in the host, and a rapidly evolving environment. Clonal interference in these circumstances leads to conditions where the greater fecundity of clones is unable to offset their poor adaptation. Sexual and clonal populations then either co-exist, or sex emerges as the more stable evolutionary strategy.

**Conclusions:**

Sex can out-compete clones in a rapidly evolving environment, such as that characterized by pathogens, where clonal interference reduces the adaptation of clonal populations and clones adopt advantageous mutations more slowly. Since all organisms carry parasitic loads, the model is of potentially general applicability.

## Background

Sexual reproduction in higher eukaryotes is an evolved adaptation that uses a well-defined mechanism for mixing DNA in progeny (segregation, meiosis, gametogenesis and syngamy). One feature of this reproductive adaptation is the emergence of males, which impose costs on sex that are not borne by clonal reproduction [1-4]. Cloning should be widely prevalent as a consequence and sex under constant threat of extinction. Instead, sex is widespread, whereas clonal species exist at the margins and, with a few exceptions, are considered to have short evolutionary trajectories [3-5]. Ridley summarised the position as follows [6]: “The spindly taxonomic distribution of asexual reproduction suggests that asexual lineages have a higher extinction rate than sexual lineages – that asexual lineages usually do not last long enough to diversify into a genus or larger taxonomic group”.

The costs of sexual reproduction were identified in two ways. Maynard Smith [1] and Williams [2] independently identified a cost of meiosis associated with the production of haploid gametes by random sampling of diploid genomes. Although fusion of haploid gametes reinstates diploidicity in the offspring, individual offspring are now no longer copies of their parents. Maynard Smith also identified a fecundity problem: the two-fold cost of males [3], (prefigured by Weismann [7]). Clonal mothers produce twice as many reproducing mothers in each generation as sexual mothers because the sexual mothers also produce males. If males and females are produced in equal numbers, the clonal mother and her progeny should rapidly drive the sexual population to extinction.

Maynard Smith’s challenge [3] is greater still if a sexually-reproducing population is to resist invasion by a sexual mother converting to clonal reproduction because, at the moment of conversion, the adaptation of the new clonal mother is the same as her former sexual self. Her fitness therefore doubles. If she survives drift, clonal invasion of the sexual population is rapid and sex is extinguished. However, invasion could be prevented if the clonal mother and her offspring suffered a rapid decline in fitness relative to a sexual mother.

The cost of meiosis and the two-fold cost of males are not the same thing. A sexual population that is genetically homogeneous will have no cost of meiosis because there can be no loss of genetic relatedness between parents and offspring. However, conversion of a sexual mother to clonality under these conditions will still extinguish sex.

There are at least four known differences between clonal and sexual populations that might account for differences in fitness under otherwise similar circumstances. First, there is a hypothetical difference in the rate of adoption of advantageous mutations by sexual and clonal populations. This difference was first identified by Kirkpatrick and Jenkins [8] (abbreviated as the KJ effect in this paper). Kirkpatrick and Jenkins argued as follows: an advantageous mutation in a sexual population sweeps uninterruptedly from one homozygous state to a new, better-adapted homozygous state in a single, seamless transition, whereas a clone is arrested at the heterozygote stage. It requires a second advantageous mutation to proceed to homozygosity. Because the target size for the second mutation is half that of the first mutation, it takes more than twice as long, on average, for clones to move from one homozygous state to the next. Sex should show an ongoing rate advantage over cloning if the external environment changes constantly to create opportunities for continuous adoption of new advantageous mutations. This is a moving optimum for the diploid population. It is an environment that was foreshadowed by Felsenstein (“gene substitutions act to counteract the effects of environmental change, as if the population were running as hard as it could to stay in the same place.” [9]). Felsenstein’s paper also introduced the concept of lag load as a measure of the mismatch between an organism and its environment.

The approach adopted by Kirkpatrick and Jenkins [8] was analytical, and relied on a simplifying assumption; namely, that in a linear string of coupled, 2-allele loci, each locus would mutate in turn, after previous mutations had swept to fixation. For a clonal locus, this requires two rates of mutation: a slower one for the first mutation that sweeps to heterozygosity, and a faster one for the second mutation to occur before another locus takes its turn to adopt the first advantageous mutation. This requirement, and the methods used to calculate heterozygote fitness, was subject to criticism [10]. The advantage to sexual reproduction also disappears when mutation rates are low, because mitotic recombination provides a mechanism, at least in principle, by which clones can adopt advantageous mutations in a single sweep, mimicking their sexual counterparts [11]. Taken at their face value, these two studies [10,11] appear largely to have excluded a rate advantage to sexual reproduction in adopting beneficial mutations and, in so doing, minimized the importance of the KJ effect.

Fisher [12] had earlier identified two other sources of advantage to sexual reproduction. In the first, two beneficial mutations occurring near-contemporaneously in separate individuals can rapidly be brought onto the same chromosome by sexual reproduction and crossing-over, whereas a clonal population must adopt the two beneficial mutations sequentially. This delays the adjustment of clonal populations to maladaptation. Muller [13] independently stated the same idea, and it is now known as the Fisher-Muller hypothesis. It requires mutations of small effect, large populations and high mutation rates for sex to acquire its advantage [14,15]. Fisher also identified a separate disadvantage to clones, which sets them apart from sexually reproducing species [12]. In polymorphic clonal populations, the polymorphism results in a series of reproductively isolated clonal lines, any one of which can receive an advantageous mutation. If a clonal line is poorly adapted, the advantageous mutation may be lost as the polyclonal population moves to monoclonality through expansion of a fitter clone, whereas in a sexual population, an advantageous mutation can switch its background through independent assortment and crossing-over. Clonal polymorphism wastes advantageous mutations more than sexual reproduction. Muller had similar ideas [13] but, importantly, also anticipated the possibility that advantageous mutations in separate clonal lines with similar fitness would cause the lines to compete with each other, with no immediate emergence of a winner. This is an early verbal version of clonal interference. The existence of clonal interference has been supported both theoretically and experimentally in clonal species such as bacteria [16]. Manning also identified the inefficiencies existing in polyclonal populations that prevent sweeping of advantageous mutations, reflecting the reproductive isolation of clonal lines [17]. No party appears to have shown that the lower adaptation of clonal populations, by whatever mechanism, is sufficient to reduce the fitness of these populations below that of equivalent sexual populations.

A fourth difference that could give an advantage to sex is the elimination of interference between coupled loci by crossing-over. This has been widely seen as conferring a major fitness advantage on sexual reproduction [18] because it allows elimination of Hill-Robertson interference. It is not clear that, in its genome-wide application, it operates rapidly enough to compensate for the fecundity advantage of clones.

Both the KJ effect [8] and the Fisher effect [12] can arguably be considered within the wider context of mutation and selection, even though Fisher’s argument was not couched in mathematical terms. Both mechanisms provide a nominal advantage to sexual reproduction in circumstances where there is acute relief of populations from maladaptation. However, acute relief, if it is erratic and infrequent, is unlikely to produce consolidation of major evolutionary adaptations such as sexual reproduction. What is needed is an environment that imposes chronic maladaptation, to which sexual reproduction can be the adaptive response. It was Hamilton and his colleagues [19,20] who suggested that parasites had the properties needed to generate this type of chronic environment, because parasites are capable of endless, rapid evolution and are frequently pathogenic. This insight was incorporated into a biotic model in which the fitness of a (haploid) host was inversely coupled to that of a (haploid) parasite. Since parasites are able endlessly to shift their genotype through a limited, but non-trivial, genotypic space, host defences likewise required constant change. Hamilton and his colleagues [19,20] assumed that a limited library of host defences was built from several di-allelic loci, whose permutation, through crossing-over, gave rise to the defence library catalogue. Negative frequency-dependent selection (NFDS), acting in turn on individual haplotypes within this library, saw some haplotypes eliminated in rolling cycles of depletion and loss through drift. The function of sex on Hamilton’s view was to reconstitute missing haplotypes through crossing over between loci. A low mutation rate was needed to sustain the di-allelism of each locus. A prediction of this model is that host allele frequencies should oscillate as defence sectors successively come into prominence and then decline, only to be reconstituted.

It is not clear that parasites, in the real world, are restricted to limited genotypic spaces. Indeed, those that have been studied extensively, such as influenza viruses, mutate across large landscapes [21-24]. Host defences, in turn, almost certainly need large allele spaces to provide defence coverage against this extensive mutation. The number of defence loci needed could be much larger than those covered by standing variation, and the overall supply could need extensive complementation by mutation. There is currently a dearth of models in which the parasite environment is characterized by NFDS and the host response is one of mutation and selection operating in a large allele space. At their simplest, mutation and selection in a static environment reflect the competing claims of stabilizing selection, which reduces genetic variation, and new mutations that tend to increase it. However, mathematical treatment of mutation/selection balance is challenging, even for this simple case [25]. It is more so when selection varies with time [26-30]. Given the historic difficulty in developing analytical methods for treating mutation/selection balance in changing environments, we chose instead to employ a computational approach.

We report on a computational approach that allows us to model mutation and selection in changing environments deploying NFDS on hosts. The approach enables us to identify two parameter spaces that support sex. The first parameter space develops under lag load, and allows sexual populations to adopt advantageous mutations faster than clonal ones. The second space develops where multiple loci are engaged in host defences, and where these defences operate at the high mutation rates needed to maintain fitness in the face of a rapidly mutating parasite environment imposing NFDS. The ensuing host polymorphism leads to clonal interference, differentially reducing the ability of clones to track rapidly changing environments and giving sex a comparative fitness advantage that allows it to compete with cloning on equal terms, or displace it.

## Results

### Recapitulating Kirkpatrick and Jenkins

**Figure 1:**
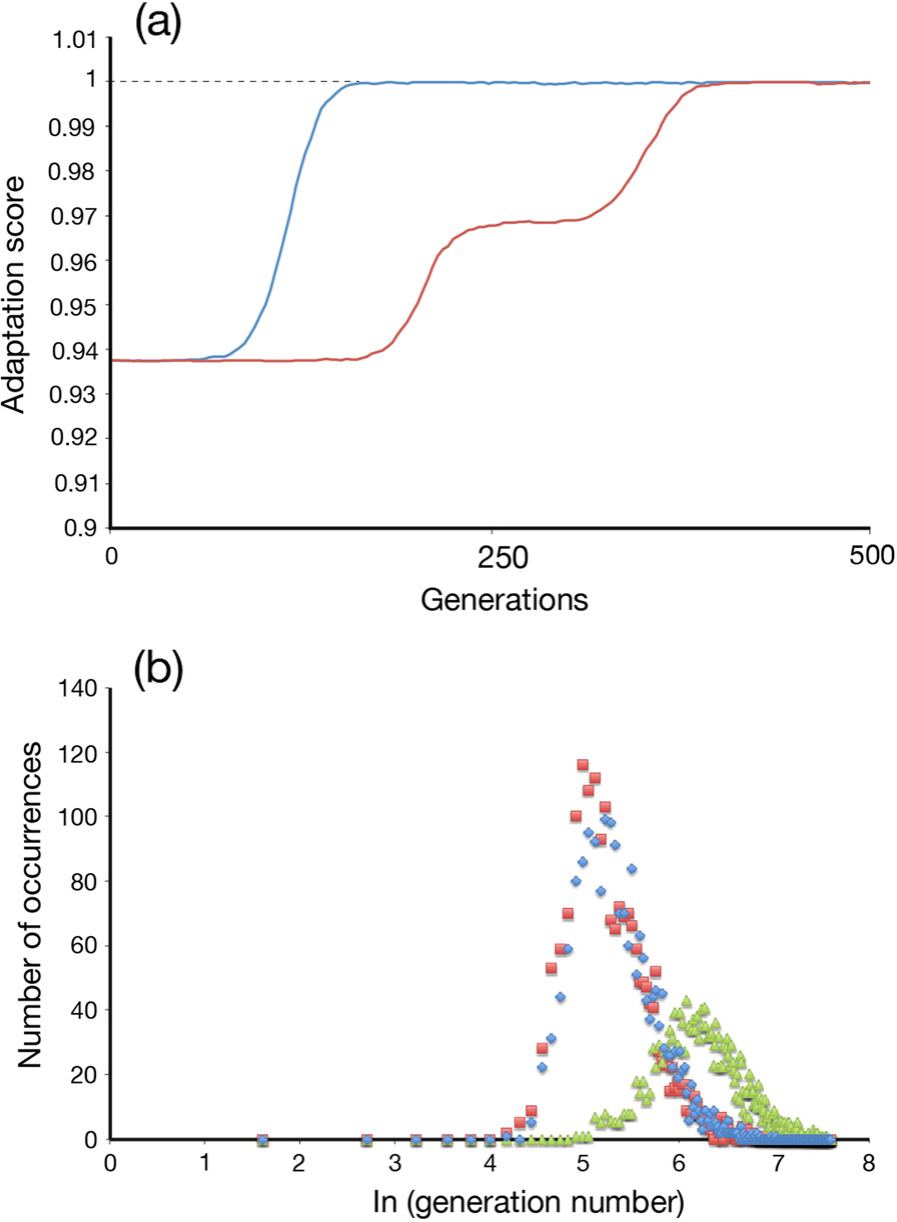
**Computer simulations of adoption of advantageous mutations. (a)** Computer simulation showing adoption of advantageous mutations at a single diploid locus. The starting populations are sexual (blue) and clonal (red); (population size, *N* = 1500; host mutation rate, *μ*_*h*_, = 10^-4^ bits/allele/generation). Each starting population is uniformly homozygotic and maladapted by one bit per allele (starting adaptation of 0.9375). The parasite environment is uniform and fixed for the duration of the simulation (that is, mutation rate, *μ*_*p*_, = 0). The sexual population shows a single sweep to full adaptation following adoption of an advantageous mutation, whereas the clonal population proceeds through two sweeps, reflecting the need for adoption of two mutations to proceed to full homozygous matching of the parasite. The target size for the second mutation in the clonal heterozygote is half that for the first mutation, halving the rate of adoption. This is the basic result shown by Kirkpatrick and Jenkins [8]. **(b)** Distribution of times (in generations) to adoption of advantageous mutation, plotted on lognormal abscissa. Means and standard deviations in this simulation are: 5.35 ± 0.54 (red) (210.7 generations); 5.39 ± 0.53 (blue) (220.0 generations); 6.35 ± 0.45 (575.2 generations).

We confirm the KJ effect [8] by computer simulation (see Methods section for the way in which the genetic algorithm is set up). In the simplest case, where the parasite is a uniform population that is held fixed, the simulation provides a 17-point numerical scale to measure the adaptive value of an individual host allele. Diploid genotypes have a 33-point scale because of the averaging of allelic adaptations. Figure 1(a) shows the response of a uniform diploid population that is maladapted by one mutation per allele in a static environment. The starting adaptation score of the diploid genotype is 1.0 - 0.0625 = 0.9375. The sexual population moves to an average adaptation score of 1.0 after adoption of one beneficial mutation, whereas the clonal population moves to an average adaptation score of 0.96875 after the first mutation, and 1.0 after the second. Figure 1(a) is the same as Figure 1 in [8] but generated computationally in real time using our simulation. Adoption of beneficial mutations in the simulation is stochastic. Figure 1(b) shows data for time to adoption plotted on a lognormal scale. The times to adoption, in generations, for the sexual population (measured at population adaptation = 0.953) are in red. Times to adoption of first mutation in a clonal population (measured at population adaptation = 0.953) are in blue; time to adoption of the second clonal mutation (measured at 0.984) in green. Lognormal distributions were Gaussian for all three curves as tested by the method of Burmaster and Hull [31]. The time to adoption of the second mutation is 2.5-3 times that of the first. This ratio is expected since the target size for the second mutation is half the size of that for the first mutation and the clock starts for the second mutation as the first mutation is adopted. There is evidence of slight differences in the effects of drift on homozygote and heterozygote populations. The slower adoption times of the second clonal mutation is general across a range of mutation rates, *μ*, and population sizes, *N*. (*N* = 300 – 10,000; *μ* = 10^-6^ – 10^-3^).

### The KJ effect with multiple loci

Kirkpatrick and Jenkins [8] adopted an analytical approach to the calculation of population fitness for sexual and clonal populations over the medium term, and made a number of simplifying assumptions. These included: (i) loci were numerous; (ii) loci were binary, i.e. 0 or 1; and (iii) loci fully adopted mutations before another locus proceeded to adoption. These, and other assumptions, are restrictive and have been noted as such [10]. We avoid the restrictions by setting mutation rates independently for hosts and parasites and allowing for stochastic adoption of mutations. We then compute all adaptation scores on-the-fly for each allele for each generation, placing no limitations on the order of mutation adoption. We also use 16-bit loci to increase allele space, and study single loci and small-to-moderate numbers of loci where the parasite environment evolves continuously to reduce the adaptation of the host through NFDS. A key relationship at steady state (defined in Methods) is the ratio of mutation rate in the parasite, *μ*_*p*_, to that in the host, *μ*_*h*_, since a function of this ratio gives the lag load, *LL* (i.e. *LL* = *f* (*μ*_*p*_/*μ*_*h*_)). As noted in the Introduction, a lag load provides opportunities for continuous adoption of advantageous mutations by the host [3,9].

**Figure 2:**
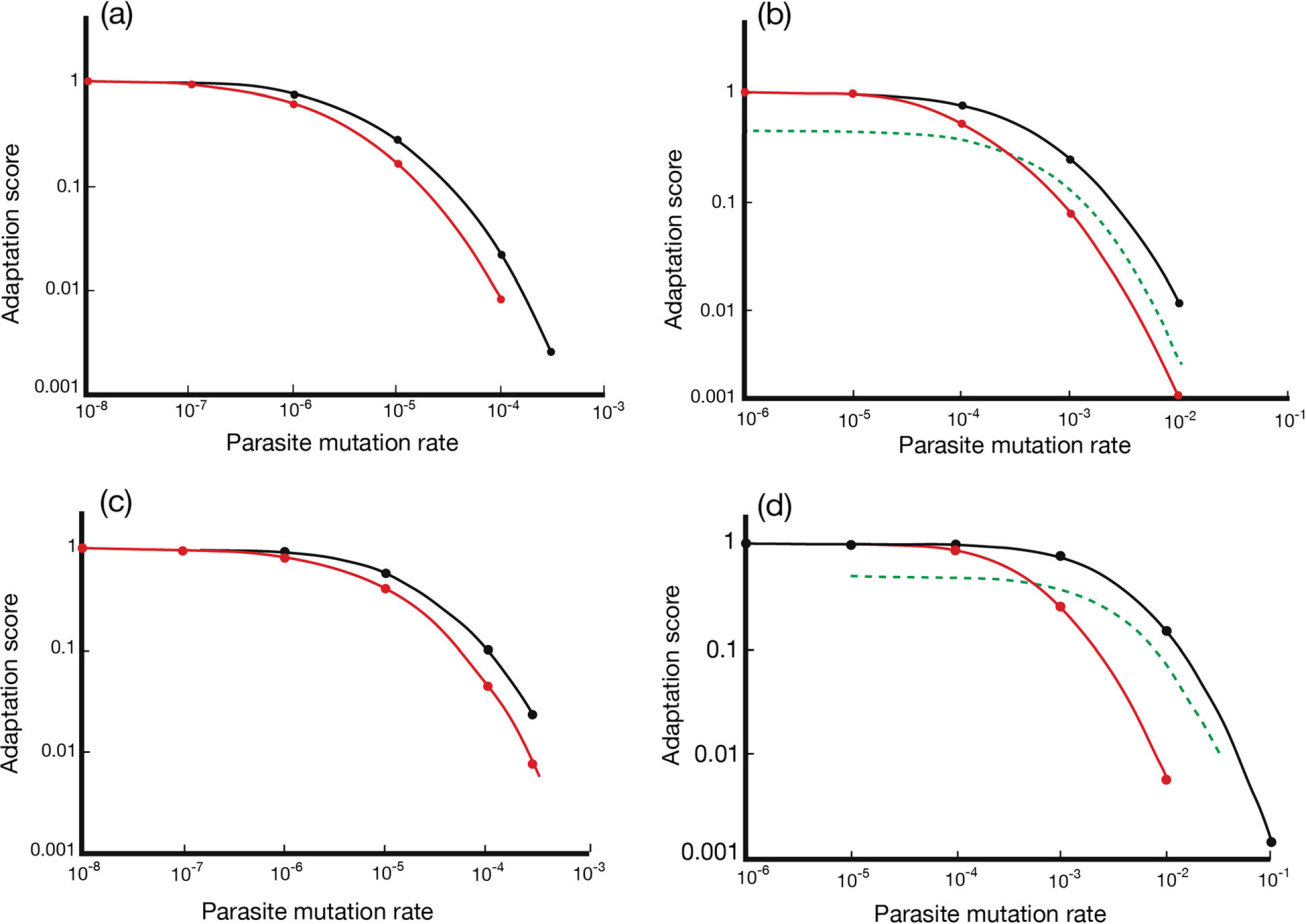
**Adaptation profiles for sexual and clonal host populations at two host-mutation rates. (a)** Population size, *N* = 100; *μ*_*h*_ = 10^-6^ bits/allele/generation; *μ*_*p*_ = 10^-8^-10^-3^ bits/allele/ generation; number of loci, *L* = 10. Black curve = sexual population; red curve = clonal population, both under the same steady-state conditions. The value of *μ*_*p*_/*μ*_*h*_ is progressively increased in each curve by increasing *μ*_*p*_ at constant *μ*_*h*_. The figure shows that the rate advantage accruing to sex in the adoption of advantageous mutations persists in temporally complex environments when numerous allelic species are in play and there is potentially more than one mutation occurring in each generation. **(b)***N* = 100; *μ*_*h*_ = 10^-3^ bits/allele/generation; *μ*_*p*_ = 10^-6^-10^-2^ bits/allele/ generation; number of loci, *L* = 10. Black and red curves as in **(a)** above. The green curve represents the adaptation score for the sexual population adjusted by a factor of 0.5 to reflect its lower fitness relative to the clonal population due to the 2-fold cost of males. **(c)***N* = 3000; *μ* = 10^-6^ bits/allele/generation; *μ*_*p*_ = 10^-8^-10^-3^ bits/allele/ generation; number of loci, *L* = 10. Black and red curves as in **(a)** above. The data are similar to those in **(a)** above but the larger population has produced a slightly larger difference in steady-state adaptation scores for sexual and clonal populations. **(d)***N* = 3000; *μ* = 10^-3^ bits/allele/generation; *μ*_*p*_ = 10^-6^-10^-1^ bits/allele/ generation; number of loci, *L* = 10. Black and red curves as in **(a)**; the green curve as in **(b)** above. The clonal population is also subject to interference with selection, due to increased polymorphism at the high mutation rate. This interference serves further to reduce the adaptation score. Standard Errors of the Mean are approximately the width of the line connecting the data points.

Data are shown in Figures 2(a-d) for a range of lag loads generated in two populations (*N* = 100, 3000) as parasite mutation rates increase at fixed host mutation rates (Figures 2(a,c), *μ*_*h*_ = 10^-6^ bits/allele/generation; Figures 2(b,d), *μ*_*h*_ = 10^-3^ bits/allele/generation). Adaptation scores decline as parasite mutation rates increase relative to those of the host (i.e. *μ*_*p*_/*μ*_*h*_ increases). The average adaptation scores of the sexual populations (black curves) are always higher than those of the clonal populations (red curves) under equivalent conditions.

**Figure 3:**
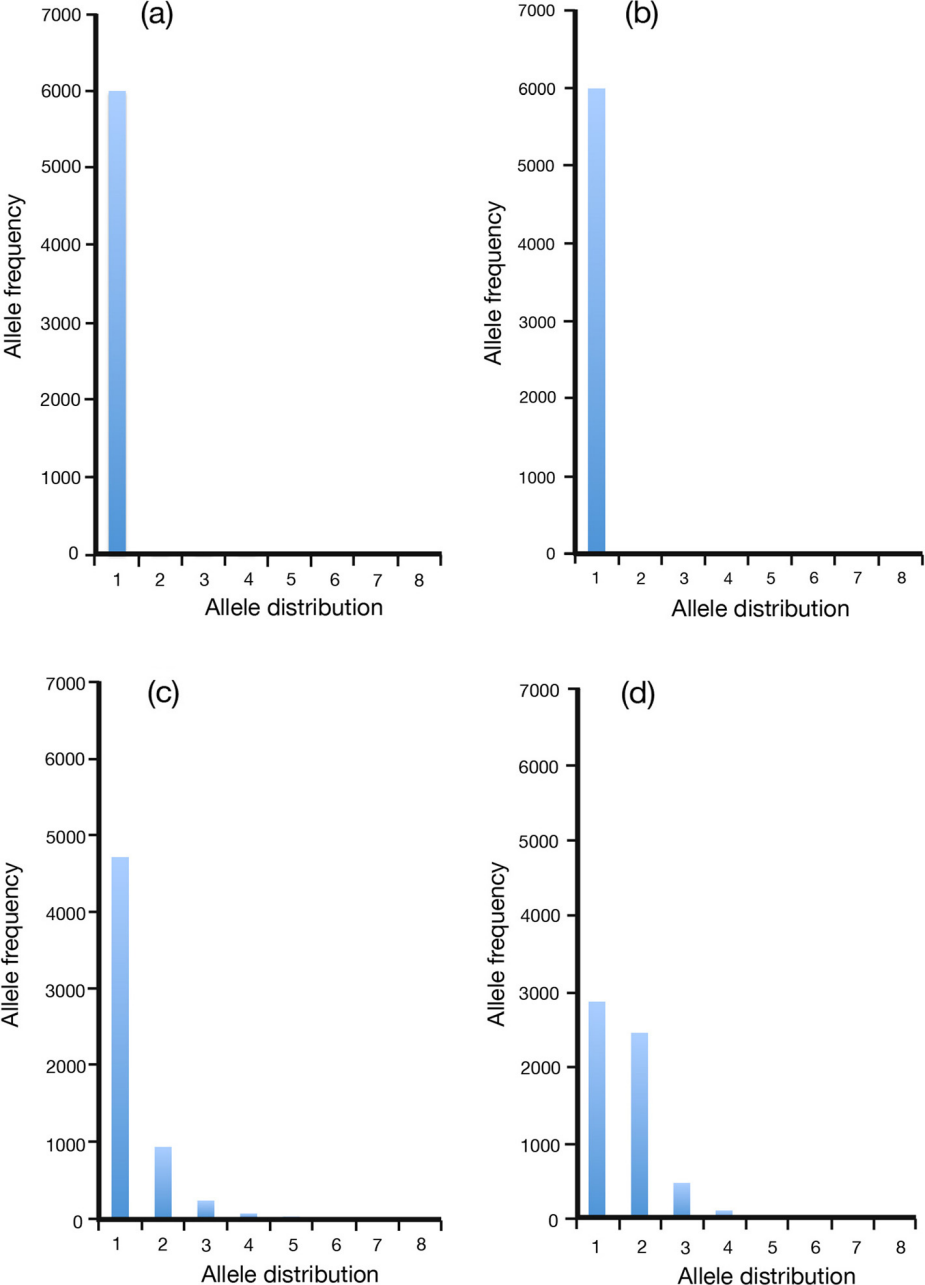
**Allele frequency distributions with and without lag load in sexual and clonal populations. (a)** Sexual population (*N* = 3000; host mutation rate, *μ*_*h*_ = 10^-4^ bits/allele/generation; parasite mutation rate, *μ*_*p*_ = 0; number of loci, *L* = 3) at steady state. The population is essentially fully adapted, and monomorphic over the short and long term. There is a small standing variation of low frequency alleles that does not register on the graph. **(b)** Clonal population (*N* = 3000; host mutation rate, *μ*_*h*_ = 10^-4^ bits/allele/generation; parasite mutation rate, *μ*_*p*_ = 0; number of loci, *L* = 3) at steady state. As in **(a)** above, the population is essentially fully adapted and monomorphic over the short and long term. Standing variation is almost identical to that in **(a)** above. **(c)** Sexual population (*N* = 3000; host mutation rate, *μ*_*h*_ = 10^-4^ bits/allele/generation; parasite mutation rate, *μ*_*p*_ = 10^-2^ bits/allele/generation; number of loci, *L* = 3) at steady state. Rank order frequency distribution (frequency average of commonest allele, second commonest, etc.) reflects the long run rank order averages from Figure 5(a). Substantial lag loads increase the frequency of lower order alleles. **(d)** A clonal population (*N* = 3000; host mutation rate, *μ*_*h*_ = 10^-4^ bits/allele/generation; parasite mutation rate, *μ*_*p*_ = 10^-2^ bits/allele/generation; number of loci, *L* = 3) at steady state. Rank-order frequency distribution of alleles reflects the long run rank order averages from Figure 5(b). Substantial lag loads increase the rank order frequency of previously rare alleles and cap the commonest allele at slightly less than 0.5. The level of heterozygosity increases markedly when compared with the sexual population under the same circumstances.

**Figure 4:**
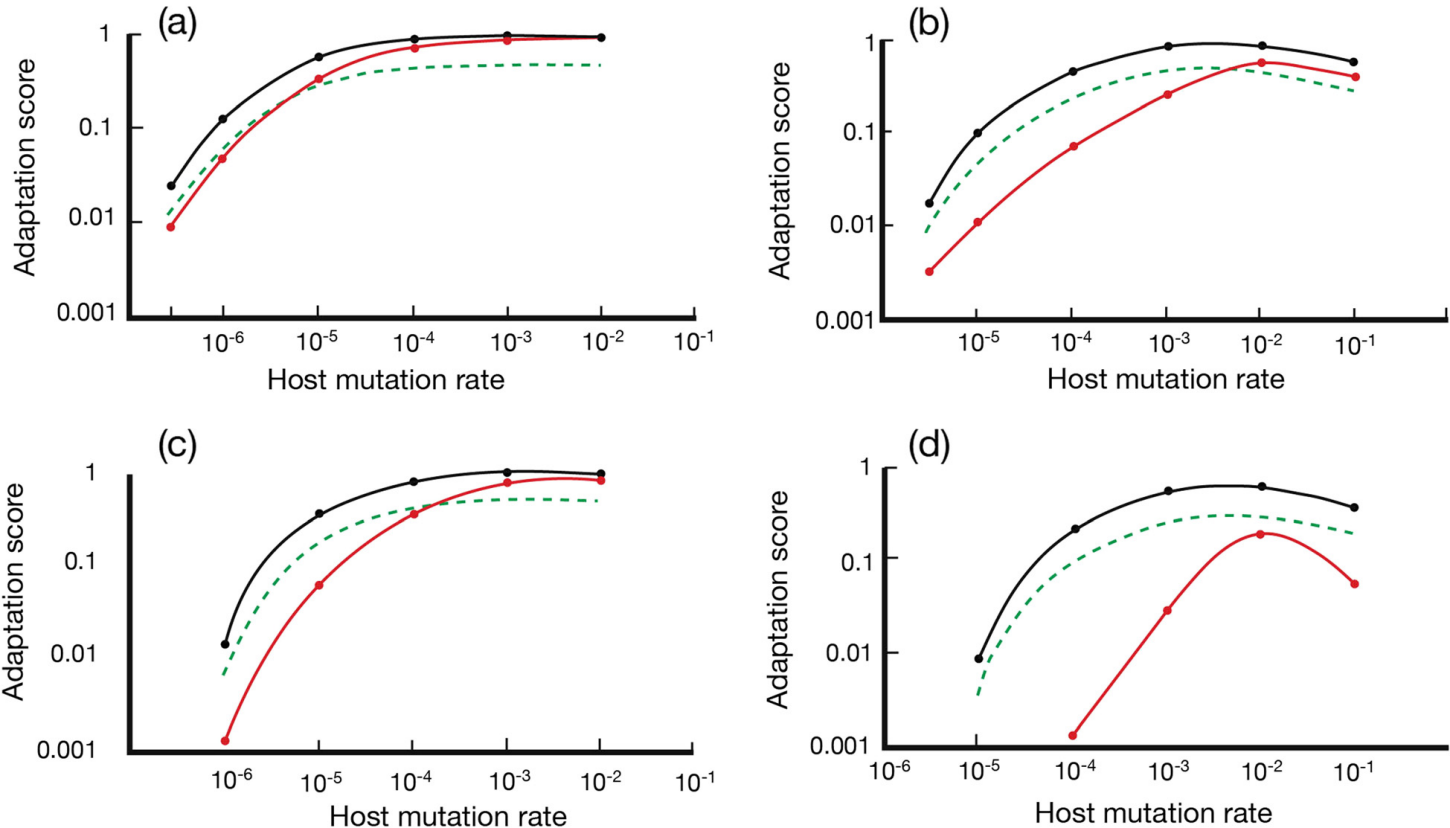
**Adaptation profiles for sexual and clonal host populations at fixed parasite-mutation rates. (a)***N* = 3000; *μ*_*p*_ = 10^-4^ bits/allele/generation; *μ*_*h*_ = 10^-7^-10^-2^ bits/allele/ generation; number of loci, *L* = 10. Black curve = sexual population; red curve = clonal population, under the same steady-state conditions. For a parasite mutation rate, *μ*_*p*_, that is fixed, hosts have optimal adaptation scores when their own mutation rates are similar to or faster than that of the parasite. Curves of relative fitness (green curve) (= adaptation scores adjusted for fecundity) show that cloning displaces sex as the populations optimize. **(b)***N* = 3000; *μ*_*p*_ = 10^-3^ bits/allele/generation; *μ*_*h*_ = 10^-6^-10^-1^ bits/allele/ generation; number of loci, *L* = 10. Black curve = sexual population; red curve = clonal population, under the same steady-state conditions. Differences in adaptation scores between clone and sex increase when compared with data in **(a)** above, an effect attributable to the effect of increased host mutation rates on levels of host polymorphism and polyclonality. The increase in polyclonality disproportionately and adversely affects the adaptation of the clonal population. **(c)***N* = 3000; *μ*_*p*_ = 10^-4^ bits/allele/generation; *μ*_*h*_ = 10^-6^-10^-2^ bits/allele/ generation; number of loci, *L* = 20. Black curve = sexual population; red curve = clonal population, under the same steady-state conditions. **(d)***N* = 3000; *μ* = 10^-3^ bits/allele/generation; *μ*_*p*_ = 10^-6^-10^-1^ bits/allele/ generation; number of loci, *L* = 20. Black curve = sexual population; red curve = clonal population, under the same steady-state conditions. The data are similar to those in (a-c) above but the combination of a larger number of loci and higher parasite mutation rate markedly suppresses the adaptation of the clonal population. Standard Errors of the Mean are approximately the width of the line connecting the data points.

The long-run steady state values of population adaptation that arise at low host mutation rates (10^-5^ or less) are largely independent of the absolute values of the mutation rates *μ*_*p*_ and *μ*_*h*_, but are a function of the ratio, *μ*_*p*_/*μ*_*h*_. When *μ*_*p*_/*μ*_*h*_ ~ 1, both sexual and clonal populations are essentially monomorphic (effective allele number = ~ 1.0), but as *μ*_*p*_/*μ*_*h*_ approaches 100 or more, sexual populations remain close to monomorphic, whereas clones become dimorphic (effective allele number = ~2.0). Supporting data is shown in Figure 3. What is seen in Figures 2(a) and (c), therefore, is a straight Kirkpatrick and Jenkins (KJ) rate advantage to sex emerging as lag loads increase (that is, ratio of effective allele number, clonal/sexual tends to 2). Large lag loads are needed because these loads indicate maladaptation, and that, in turn, increases the target size for adoption of advantageous mutations at a particular locus. This dilutes the chances that an advantageous mutation will convert a clonally heterozygote position to homozygosity. The progressive loss of lag load sees a reduction in the size of the mutation target and an increase in the chance of heterozygotes converting to homozygotes (reflected in trend of effective allele number to 1). When host matching of the environment reaches a maximum, and lag loads are very small, the lower fecundity of sexual reproduction greatly reduces its fitness relative to clones. The data in Figure 2(c) show an adaptation advantage to sex of ~2.3 when *μ*_*h*_ = 10^-6^ and *μ*_*p*_ = 10^-4^, which attenuates as *μ*_*p*_ progressively declines. Figures 2(b) and (d) show a second effect emerging as host mutation rates are increased. For a host mutation rate in Figure 2(d) of 10^-3^, the parasite mutation rates needed to produce the same lag loads as *μ*_*p*_ at 10^-4^ in Figure 2(c) are 10^-2^ for sex and ~3 x 10^-3^ for clones, an ~10-fold reduction in *μ*_*p*_/*μ*_*h*_ for sex and ~35-fold reduction of *μ*_*p*_/*μ*_*h*_ in clones. At the same time, the number of alleles rises to 18.9 for the sexual population and 20.2 for the clonal, from 1.00 and 2.01 respectively when *μ*_*h*_ = 10^-6^ under similar lag loads. This large increase in polymorphism with increase in host mutation rate markedly interferes with clearance of maladapted alleles in clonal populations. We return to this point in presenting the data that underpins Figures 4(a-d).

So far, we have shown data for adaptation scores. Fitness, in this simulation, is a multiplicative function of adaptation and fecundity, and the two-fold cost of males in sexual reproduction is reflected as a two-fold fecundity difference between clonal and sexual populations. The green curves in Figures 2(b) and (d) represent the fitness of sexual populations relative to clonal populations obtained by halving the adaptation scores of sexual populations; clonal fitness is represented by the solid adaptation curve in red, since the fecundity factor is 1.0 relative to 0.5 for sexual reproduction. These data show that sexual populations are less fit than clonal ones at small lag loads, but a transition point exists past which sexual fitness exceeds clonal fitness. This transition point represents a parasite mutation rate, *μ*_*p*_, at which, for a given host mutation rate, *μ*_*h*_, the parasite environment causes an equal loss of fitness in both populations; above this parasite mutation rate, clonal populations are less fit than sexual ones. Importantly, this cross-over point sits within a not-implausible range of lag loads. When the same approach is adopted for data in Figure 2(a), there is effectively no cross-over point, and for data in Figure 2(c), it applies at relatively high lag loads. We conclude from these data that the KJ effect at low mutation rates, both *μ*_*p*_ and *μ*_*h*_, allows sexual populations to go a considerable way in matching the fitness advantage of clonal populations, subject to the existence of adequate lag loads.

### Emerging adaptation differences: understanding the mechanism

**Figure 5:**
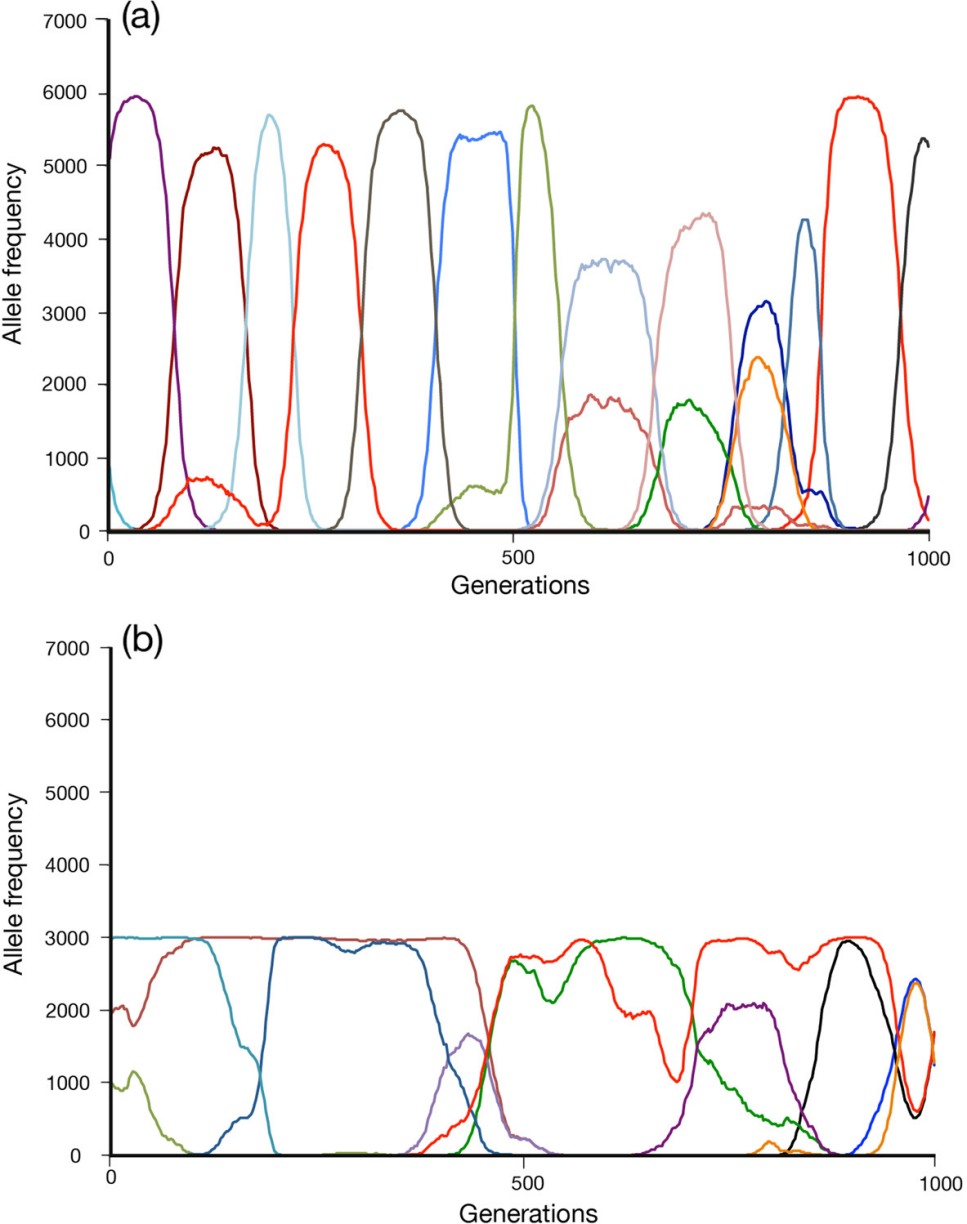
**Allele behaviour of sexual and clonal populations under lag load. (a**) A sexual population (*N* = 3000; host mutation rate, *μ*_*h*_ = 10^-4^ bits/allele/generation; parasite mutation rate, *μ*_*p*_ = 10^-2^ bits/allele/generation; number of loci, *L* = 3) at steady state. The population adaptation score for each locus is an average of 0.726, and the overall population adaptation score is 0.382. Allele frequencies here, and for **(b)** below, are for those alleles whose maxima exceed a frequency of 600. Different colours represent different allele species. Most alleles do not cycle more than once but there are occasional instances of bimodality, indicating a temporary arrest of a parasite allele in allele space. **(b)** Clonal population (*N* = 3000; host mutation rate, *μ*_*h*_ = 10^-4^ bits/allele/generation; parasite mutation rate, *μ*_*p*_ = 10^-2^ bits/allele/generation; number of loci, *L* = 3) at steady state. The population adaptation score for each locus is an average of 0.564, and the overall population adaptation score is 0.181. Maximum allele frequencies are capped at 3000 under substantial lag load for reasons discussed in the body of the paper, making the clonal population fully heterozygotic under these conditions. High-frequency alleles, capped at 3000, show much longer lifetimes than their sexual counterparts, due to interference with selection. An example of this interference is the fluctuation in allele frequency associated with very long-lived alleles (illustrated by the red line), where partial dips in frequency followed by recovery indicate early negative selection on the genotype in question, followed by an advantageous mutation at one of the other coupled loci. This brings the genotype as a whole under positive selection, reversing the impending early loss of the allele whose frequency is shown in red. Final elimination of this allele runs beyond the end of the simulation.

In this section, we provide data that show how, in part, the effects described previously arise. First, we extract information from the simulation about allele lifetimes and allele frequencies during the course of a simulation. For technical reasons related to the tractability of computation, these data are derived from diploid genotypes with three loci, each of 2 × 16 bits. Figures 5(a) and (b) show typical profiles for sexual and clonal populations, respectively, under middle-range lag loads (adaptation scores for individual loci: sex, 0.707; clone, 0.548) using mutation rates, *μ*_*h*_, per locus of 10^-4^ bits/allele/generation for hosts, and 10^-2^ bits/allele/generation for parasites; *N* = 3000. Sexual reproduction at these mutation settings is characterized by a series of short-lived allele sweeps that mostly move towards temporary homozygosity (effective allele number = ~2). Occasionally, the population supports two alleles simultaneously at any given locus. Lowering mutation rates without shifting the ratio, *μ*_*p*_/*μ*_*h*_, between parasite and host extends allele lifetime and effective allele number tends to 1.0. In all cases, alleles come into existence and then die, rather than cycle. This behaviour reflects the size of the allele landscape being used. The non-cycling behaviour of these alleles is in contrast to those cited in [20].

Clonal populations show significantly different allele behaviour under the same conditions. Here, maximum allele frequencies are capped at 0.5 as lag load develops and effective allele number tends to 9.0. As with sexual reproduction, lowering mutation rates without shifting the ratio, *μ*_*p*_/*μ*_*h*_, extends allele lifetime without lifting the frequency cap. We argue the explanation for the emergence of the cap is as follows. An average clonal adaptation score of 0.55 per locus (see previous paragraph) represents a mismatch of approximately 14 bits for a diploid locus of 2 × 16 = 32 bits. If the one-bit components of the locus are homozygous (either 0,0 or 1,1) and adopt an advantageous mutation, they move to heterozygosity (0,1 or 1,0). The next advantageous mutation to undergo adoption by the locus has no better than a 1-in-14 chance of occurring at the heterozygotic bit in question. The chance of adoption is actually lower than that, as we show shortly, because alleles start losing adaptation as soon as they have been adopted. Taken together, the low chance of adopting a second mutation at the heterozygote locus and the deterioration in the adaptation of its carrying allele make its chances of moving to homozygosity effectively zero, and this is borne out by the data. The cap on the 0.5 frequency rises as the parasite mutation rate is progressively lowered (through 10^-3^, 10^-4^, 10^-5^, etc.) relative to the host (that is, as the value of *μ*_*p*_/*μ*_*h*_ falls, because the average adaptation score of the clonal population increases, as does the chance of adopting a heterozygote-converting advantageous mutation.

Histograms of allele frequency distribution are shown in Figure 3. At the low *μ*_*p*_ of 10^-6^ bits/allele/generation, with *μ*_*h*_ = 10^-4^ bits/allele/generation, the allele distributions of sexual and clonal populations are essentially identical, and both populations are almost entirely homozygotic (that is, effective allele number is close to 1.0). This reflects the effect of stabilizing selection in a near-constant environment. When the environment changes more rapidly, the average adaptation of the population falls. The precise level of the fall depends on the nature of the environmental change. A random walk has almost no effect, whereas NFDS imposes a lag load whose size depends on the ratio *μ*_*p*_/*μ*_*h*_ and the mode of reproduction (sexual or clonal). Where significant lag loads emerge (using *μ*_*p*_ of 10^-2^ in this particular case), there is capping of the highest clonal frequency. This result is an inevitable consequence of the KJ effect under significant lag load. It has the important effect of enforcing greater polymorphism (strictly, a higher effective allele number) on the clonal population than the sexual population, thereby creating a more substantial opportunity for negative interactions between co-alleles at single loci. The effect is independent of absolute mutation rates, but rests on a suitable value of *μ*_*p*_/*μ*_*h*_.

### Allele lifetimes and declines in adaptation scores

**Figure 6:**
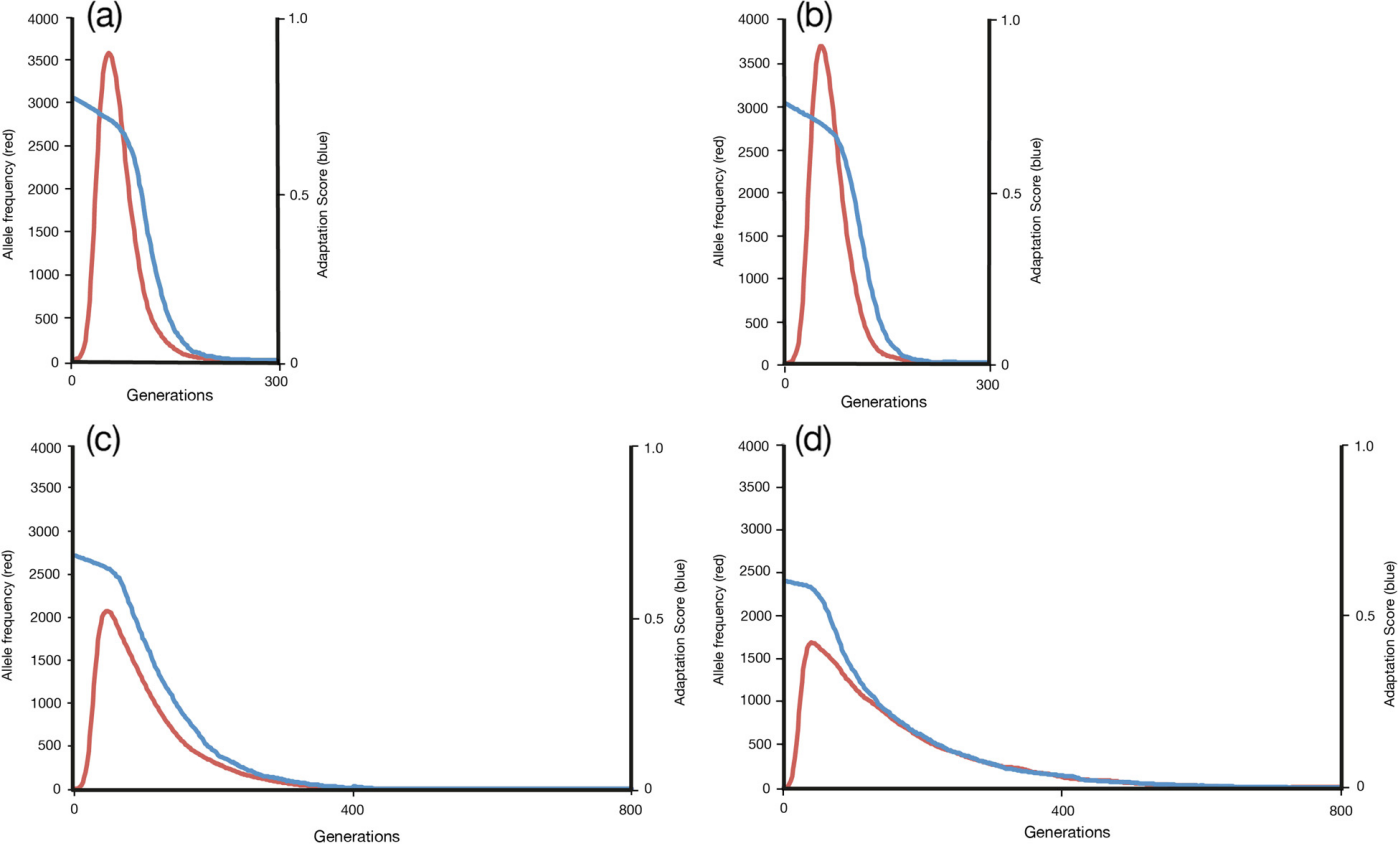
**Allele frequencies and adaptation scores as a function of time. (a)** A sexual population (*N* = 3000; host mutation rate, *μ*_*h*_ = 10^-4^ bits/allele/generation; parasite mutation rate, *μ*_*p*_ = 10^-2^ bits/allele/generation; number of loci, *L* = 1) at steady state. Allele frequency reflects composite time course for 500 adopted mutations with a frequency > 600 (= 10% of maximum possible frequency). Curves are synchronized for first appearance of the advantageous mutation. Adaptation is also scored from point of adoption of advantageous mutation. A negative frequency-dependent response begins immediately, but loss of adaptation picks up sharply after peak allele frequency has been attained. **(b)** Sexual population, with conditions and parameters as in **(a)** above, except that number of loci, *L,* = 3. The curve is similar to that in **(a)** above, showing limited effects from three loci. Data are extracted from the simulation used in Figure 3(a). **(c)** Clonal population, with conditions and parameters as in **(a)** above. The frequency peak height is now much lower than that in **(a)**, reflecting capping of allele frequency in clones under substantial lag load. The adaptation score initially declines in a similar manner to **(a)** but long tails persist in both frequency and adaptation scores, showing persisting difficulty in clearance even when interference is at single loci. **(d)** Clonal population, with conditions and parameters as in **(b)** above. In this 3-locus simulation, there is significant inter-locus interference in addition to intra-locus interference, with lower peak frequency than **(c)** and longer tails for losses of allele frequency and adaptation. Data reflect composite time-course data used in the simulation shown in Figure 3(b). Standard Errors of the Mean are approximately the width of the line connecting the data points.

Here we present the data that provide evidence of greater relative interference with selection in clones as the number of loci increase. These data fall into two sections. First, we compare average allele lifetimes in sexual and clonal populations, where there are either one or three loci in contention (Figures 6(a-d). The data for the sexual populations show effectively no difference between the distribution of allele lifetimes at a single locus (Figure 6(a)) and three loci (Figure 6(b)), nor rates of decline of adaptation scores. Figure 6(c) shows the average allele lifetime behaviour for a single clonal locus under the same conditions. The maximum allele frequency is much less, due to the capping effect at significant lag loads, and there is already evidence of interference with selection, as evidenced by the long tail for allele frequency against time. The rate of decline of the adaptation score is also slower. We know from the data on single loci in sexual reproduction that declines in adaptation score can be much faster. We attribute the slower decline in clonal reproduction to increasing polymorphism that, in turn, spreads the effects of NFDS across a larger number of different alleles. This reduces differences in selection pressure on different clonal genotypes and slows elimination of individual allele species. The argument is supported by the lower adaptation score in clonal alleles on starting, which indicates that the parasite population already has wider surveillance of the host population than its equivalent in the sexual populations. As a consequence, selection pressure on individual alleles is reduced and the effect of polymorphism is enhanced further. The slower timeline of the decline in adaptation score also increases the likelihood that a novel advantageous mutation will occur in an allele of declining adaptation, with a co-allele whose adaptation is also declining. These data support the existence of interference between alleles at single clonal loci.

Figure 6(d) shows the effects of coupling in a clonal three-locus genotype. This extends further the average lifetime of alleles and increases the effective allele number. The longer tail when compared with Figure 6(c) is due to a combination of intra-locus and inter-locus interference with selection, and greater spreading of the clonal targets facing parasites deploying NFDS. The starting adaptation score is now lower than in Figure 6(c) because inter-locus interference has increased effective allele number still further. The obligatory coupling that occurs in clones increases the probability that a beneficial mutation will occur in a poorly adapted background. The effect increases with the number of loci that are coupled, because overall effective allele number increases as a power function of the effective allele number at single loci, as well as contributing additional interference. The existence of multiple loci has relatively little effect in sexual reproduction if the loci segregate independently. This underpins Fisher’s explanation for the relative advantage of sex [12].

The data also show that the average initial rate at which clonal alleles sweep is essentially the same as sexual alleles for those alleles that survive drift. This addresses and eliminates another possible source of difference between sexual and clonal reproduction.

### Optimizing host mutation rates and the effects of polymorphism and locus number

This section describes the outcome when data, used in Figure 2, on the interaction between host and parasite mutation rates, are redrawn in Figure 4 to show the response of host populations to fixed parasite mutation rates. We extend these data to include examples of the effect of locus number.

All four graphs in Figure 4 show that host populations have higher fitness when their mutation rates approach those set by the parasite. These data confirm that parasites that mutate rapidly require high rates of mutation in host defences if host populations are to optimize their own fitness. Figure 4(a) shows that clonal and sexual populations progressively approach an adaptation score that is close to unity for both populations as their mutation rates rise. There is a small mutational load at these mutation rates under the conditions of the simulation, and this mutational load can be increased as selection is relaxed. The difference in adaptation scores between clonal and sexual populations at large lag loads is attributable to the Kirkpatrick and Jenkins (KJ) effect [8], covered in previous sections of these Results.

Figure 4(b) shows the effect of increasing parasite mutation rate by 10-fold. The host now shows adaptation optima at higher host mutation rates, as expected. The increased gap that emerges between clonal and sexual population fitness can be attributed, in the first instance, to higher polymorphism and an increase in clonal interference. However, as already discussed, the parasite NFDS response becomes spread over a number of genotypes as polyclonality increases, and this spread reduces differences in selective pressure on genotypes and, indirectly, any given allele. It slows the ability of the clonal population to respond to high parasite mutation rates.

When the number of host loci is increased at the lower parasite mutation rate used in Figure 4 (10^-3^), there is a small difference in maximum adaptation scores between clonal and sexual populations that is consistent. Intermediate parts of the curves show a greater disparity in adaptation between sexual and clonal populations than in Figure 4(a). In both cases, the gap is likely to reflect the emergence of clonal interference and its consequences for NFDS.

Figure 4(d) illustrates the outcome with a higher mutation rate (10^-2^) and a larger number of loci (20). There is a marked collapse in the adaptation scores of the clonal population as effective allele number rises. The KJ effect seems likely to be a significant contributor at large lag loads, where maladaptation is substantial. The picture is likely to be more complex where host populations are better adapted because of higher host mutation rates. These higher rates, in clones, would be expected to produce greater polymorphism, higher levels of heterozygosity, and weaker selection on different genotypes. Whatever the reason, sexual reproduction is now fitter over the entire range of host mutation rates.

### Testing Maynard Smith

**Figure 7:**
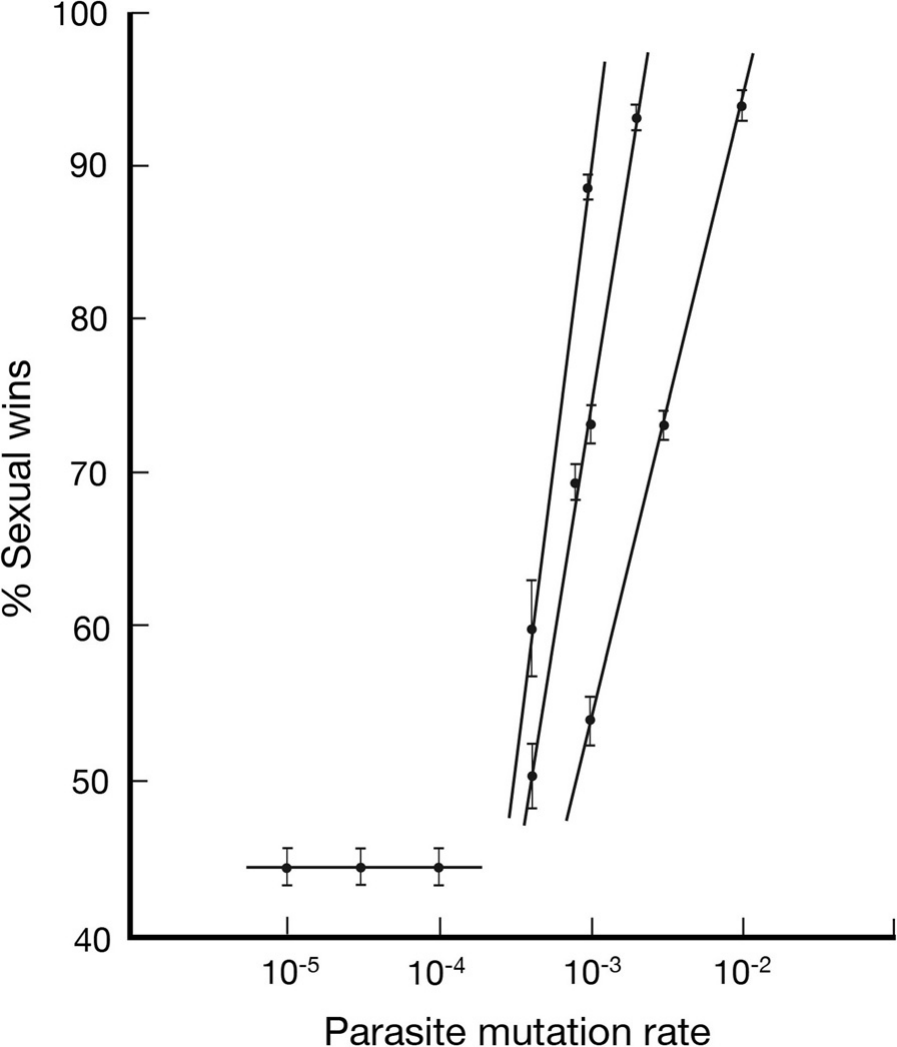
**Response of sexual populations to injection of clones.** Sexual populations (*N* = 10^2^ (i), 10^3^ (ii), 10^4^ (iii)) under lag load are allowed to stabilize at steady state rates of parasite and host mutation (*μ*_*p*_/*μ*_*h*_ = 1, *L* = 10). A constant value for *μ*_*p*_/*μ*_*h*_ is an attempt to generate similar lag loads at all data points. After stabilization, a sexual female of the highest adaptation score is converted to clonality and given a relative fecundity of 2. Because the highest adaptation scores are present only in new advantageous mutations that are still rare, the parasite population develops a negative-frequency dependent response as the frequency of the clonal allele rises. If the parasite mutates rapidly, and the sexual population is under significant lag load, the original host clone and her descendants are progressively rendered unfit and are extinguished by sex. As the parasite mutation rate, *μ*_*p*_, is reduced in *μ*_*p*_/*μ*_*h*_ (with concomitant reduction in *μ*_*h*_), the rate of loss of clonal fitness is lower during expansion of the clonal population, and clones outcompete sex. Whether sex wins or clones win depends on population size and *μ*_*p*_, for constant *μ*_*p*_/*μ*_*h*_. The three lines in this Figure show that large populations are more effective at preventing clonal invasion than small ones, as expected. All three populations show thresholds of ~45% for *μ*_*p*_ below which sex only succeeds because some clonal mothers are eliminated by drift immediately after conversion of the sexual mother. Errors are Standard Errors of the Mean.

Maynard Smith illustrated the two-fold cost of males in sexual reproduction using an example in which a sexual mother was converted to a clonal mother by mutation [3]. The effect of the conversion is that the clonal mother, immediately after conversion, has the adaptation she enjoyed as a sexual mother, but now has twice the fecundity, and therefore twice the fitness. Left to her own devices, she and her descendants will rapidly outbreed the sexual population and drive it to extinction. We have shown in simulations of separate sexual and clonal populations that a clonal population can experience a much greater loss of adaptation than a sexual population under the same conditions, and this disparity provides a mechanism by which sex can, in principle, prevent invasion by clones. In our computational model, the clone is chosen from the pool of best-adapted sexual mothers. This gives the clone an adaptation score that, at steady state, is far higher than a clonal mother would normally enjoy. The outcome of inserting a clone is an empirical matter, however, and rests on the rate at which the parasite environment causes loss of adaptation in the host. Too slow a response from the parasite and the clonal population expands rapidly at little cost. Figure 7 shows data for three populations (*N* = 10^2^,10^3^,10^4^), where the numerical value of *μ*_*p*_/*μ*_*h*_ (= 1.0) is sufficient to give sex a fitness advantage under group selection at steady state. At least two predictions follow from this brief outline. First, the parasite mutation rate, *μ*_*p*_, needs to be above some threshold that causes rapid deterioration in the environment, and second, the outcome should show some sensitivity to population size, since larger populations provide for a larger number of generations for NFDS to extinguish the descendants of a clone. Because the sexual female chosen for conversion to a clone is the best adapted, it inevitably means she has just been introduced into the simulation; she is rare and without a negative frequency-dependent response from the parasite. This frequency-dependent response has to develop quickly if the clone and its descendants are to undergo elimination. Larger host populations provide a greater number of generations within which to generate this negative frequency-dependent response. Both these predictions are borne out by the data in Figure 7.

## Discussion

The challenge for any theory of obligate bi-parental sex is to show that there are biologically relevant conditions under which individual selection in diploid sexual populations may lead to higher population fitness than in clonal ones and, further, to demonstrate that sex can hold its own or beat cloning in mixed sexual and clonal populations using fitness-dependent selection of individuals for reproduction.

We chose to pursue the idea that sex might have an advantage in dynamic environments that changed sufficiently to create differential effects on mutation and selection between clonal and sexual populations. More specifically, we were interested in environments, such as those produced by parasites, where there are ongoing adverse effects due to NFDS on hosts. Hamilton and colleagues were the first to put forward the idea that sex is an adaptation to resist parasites [19,20], and they developed a simple computational model for recombination and selection in haploids. It is not clear that this model overcomes the two-fold cost of males. We felt it would be of interest to test NFDS in a mutation-selection model where the two-fold cost of males clearly emerges.

Mutation-selection models have proven challenging to analyse [25], particularly where the environment is changing (see the extensive treatment in [27], Chap. 4). Environmental change can take several forms. It can take the form of directional selection, in which a step-jump change occurs in the environment. There is an environmental random walk that intermittently imposes directional selection but, equally intermittently, relaxes it. There is fluctuating selection, which is semantically ambiguous. It frequently means oscillatory movement, possibly erratic, between two environmental states (+/−) but could include random walking. And there is NFDS, which is effectively an ongoing series of environmental step-jumps that maintain lag load. It is not clear that any theoretical treatment, to date, treats the consequences of NFDS on a diploid population. Rather than develop an analytical approach, we chose instead to explore the possibility that a simple genetic algorithm, in the sense used in the context of Artificial Intelligence [32], could provide an adequate framework to study sex in the context of mutation and selection.

Any genetic algorithm is *sui generis*. The computations described in this paper are, in the limit, nothing more than descriptions of the behaviour of 16-bit number strings under certain assumptions. Utility lies in their ability to throw light on the behaviour of mutation/selection systems in shifting environments and to argue they occur in the maintenance of sex. The current model captures a number of expected features. These include: (i) recapitulation of the rate effect first identified by Kirkpatrick and Jenkins [8]; (ii) expected birth and death of alleles in sexual and clonal reproduction, together with plausible qualitative behaviour of individual alleles; (iii) qualitatively plausible behaviour for allele distributions, moving from dominance of stabilizing selection to effects of directional selection as environmental mutation rate increases; (iv) predicted qualitative behaviour of increasing mutation rates and locus number in hosts; (v) predicted qualitative effects of injecting fit clones into sexual populations; (vi) emergence of conditions under which sex and cloning can plausibly be in fitness balance; and (vii) the behaviour of newly formed alleles under conditions of drift, and their occasional escape from drift into the body of the population where they come under selection.

If we accept, for the purpose of argument, that the adopted computational approach has some utility, we can use it to explore more widely the behaviour of populations under NFDS. What emerge are two sets of conditions that support sex. One can be seen in an environment of substantial lag load. This load can be generated at a wide range of absolute mutation rates, provided the diploid retains a substantially sub-optimal mutation rate. The advantage to sex is due to the effect identified by Kirkpatrick and Jenkins for a single locus [8] but with this important difference: the computational approach shows the KJ effect persists in stochastic situations in mixed clonal populations, where clonal lines containing loci that would benefit from adoption of novel mutations co-exist with heterozygotic loci awaiting further advantageous mutations. As expected, the advantage disappears when the lag load disappears.

The second environment that supports sex is one that emerges conjunctively as both the number of loci under independent selection and host mutation rates increase. This environment emerges even when host mutation rates are at their optimum and reflects the emergence of a new cause of lag load that adversely affects clonal more than sexual reproduction. One direct consequence of higher mutation rates is the potential for extensive host polymorphism if clearance of deteriorating alleles from a population, sexual or clonal, is not efficient. This efficiency can vary greatly between sexual and clonal populations. At one end of the clonal spectrum, we have mutations of major effect that dominate the fitness of the clone they are adopted by. The clone displaces its rivals because of its higher fitness and the population becomes monoclonal. There is little wastage of advantageous mutations if sweeps are complete, and cloning suffers no major adaptive disadvantage relative to sex because its greater fecundity ensures clones have higher fitness. At the other end of the spectrum, advantageous mutations are of small effect, and land in numerous clonal lines. Allele clearance is poor because of clonal interference, and the numerous clonal lines that emerge each become recipients of a smaller fraction of the available advantageous mutations. The NFDS response becomes diffuse, leading to weak selection on clonal genotypes, slow declines in allele fitness, and extended allele lifetimes (Figure 6d). Sexual populations, by contrast, clear alleles of declining adaptation much more efficiently because they operate in an environment where the NFDS response remains tightly focused. Clonal populations, as a whole, lose fitness disproportionately. Ironically, this is because they carry too much variation.

This account has elements of Fisher’s explanation for sex, outlined on p.123 of his book [12], and Muller’s, outlined in his 1932 paper [13]. Both Fisher and Muller required clonal polymorphism to waste advantageous mutations, but Muller was more explicit about a parallel role for clonal interference. Both explanations were verbal. Neither Fisher [12] nor Muller [13] was apparently aware of the two-fold cost of males identified by Maynard Smith [3]. In the absence of this cost, only modest differences in adaptation between sexual and clonal populations would be required to give sex an advantage. We know now that the cost of males is substantial. It is not easy to generate polymorphism or a high effective allele number in a static or slowly changing environment because populations, both sexual and clonal, remain largely monomorphic (Figure 4 (b)); less fit outliers, generated by mutation, are efficiently removed by stabilizing selection. This is where the Red Queen enters. By changing the type of environment to one that mutates rapidly and attacks other species using NFDS, substantial polymorphism can emerge, and when it does so, it has the capacity to generate rate-related responses in target species that are not seen at low rates of change in the absence of NFDS. These conditions leave evidential traces in the real world, to be discussed shortly.

We saw from the data shown in Figure 4 that, at a high environmental mutation rate, host populations, both sexual and clonal, have their highest adaptation scores when their own mutation rates are approximately commensurate with those of the environment. Does a form of the KJ effect still operate under these conditions? The answer, in our view, is yes. There are obvious loads carried by both populations in these circumstances and these loads are disproportionately larger for clones. The clonal lines provide ample opportunity for adoption of advantageous mutations, but moving beyond the heterozygote at any one locus is next to impossible. Sexual reproduction, by contrast, allows fuller sweeps and more efficient elimination of maladapted alleles. These differences emerge when clonal and sexual populations are examined separately, but will be more pronounced in mixed populations undergoing fitness-dependent selection. The reason is that, in a population taken as a whole, the clonal population disproportionately occupies the lower end of the fitness spectrum, and is therefore disproportionately eliminated. If truncation is sharp, for example, occurring at an externally set fitness level, then small changes to parasite mutation frequency above a certain level could eliminate the clonal population entirely with little additional decline in the fitness of the sexual population.

If this reasoning is correct, then we can see that Muller may well have been correct, in a general sense, in arguing that sexual reproduction was a mechanism for evaluating the merits of numerous genotypic combinations more efficiently than cloning [13]. However, mutation rates in the genome overall are much too low to accommodate the high mutation rates that are needed to sustain sex on our computational model. It follows that there has to be a two-tier genome at the mutational level for our approach to work, and this entails focusing on interference at a small number of loci where, on a pure segregation model, sexual reproduction gains a major advantage by eliminating the interference between loci that occurs in clones. If we allow these loci to stand as proxy for chromosomes, then sex gains a major advantage by allowing the shuffling of chromosomes at each generation, allowing rapid evaluation of fitness differences between homologues. Crossing-over then becomes a further refinement in the evaluation of locus fitness through the shuffling of chromosomal sections into novel combinations that allow further dissection of fitness differences. There appears to be limited literature on differences in homologous chromosomal fitness but one striking demonstration exists in *Drosophila melanogaster*[33].

It is in the light of lower clonal fitness that one can usefully re-examine the assumptions underpinning much of the theoretical treatment of sexual reproduction. To take just one example [4], it is possible to see that the inferior properties of clonal populations under certain conditions have not been factored in as a possible source of major fitness differences between sexual and clonal reproduction.

At this point, we need to ask whether either of the scenarios identified using the current computational approach are remotely plausible. On the issue of lag loads, we simply do not know whether the size of non-optimal lag loads is consistently attainable through slow adaptation of populations. The alternative is sustained loads that represent the optimum obtainable response to a changing biotic environment, over whose rate of change hosts have no control. Hosts then have the demanding task of evolving mutation rates in their defences that are high. This evolution progressively incurs a crippling polymorphism in clonal populations as the number of loci increases, and sexual reproduction has its chance. Here we turn to the biological data.

### Experimental evidence

Probably the single most important conclusion developed in this paper is that sexual and clonal populations show markedly different fitness responses when the biotic environment requires them to mutate rapidly. A prediction is that clonal populations would be much more polymorphic than their sexual counterparts. If clearance of alleles is highly efficient in sexual reproduction, there may be little biological evidence of the mechanism at work in obligately sexual species. However, there may still be inefficiencies that leave traces of high mutation rates, particularly at loci that are implicated in defences against parasites. Thus, the extensive polymorphism of the vertebrate MHC locus (= Major Histocompatibility Complex, or HLA (Human Leukocyte Antigen) in humans)), which is well catalogued [34-36], is supportive evidence (although current catalogues have been greatly swollen by the rapid expansion of the human population). Recent evidence from a genome-wide scan suggests that there are, conservatively, 125 regions of long-lived balancing selection shared between humans and chimpanzees, in addition to the MHC [37]. In six cases, there is evidence of ancestral polymorphism. There is also evidence that the identified genes have evolved in response to pressures exerted by human and chimpanzee pathogens. Similarly, there is recent evidence of ancestral polymorphism and ongoing balancing selection at disease resistance loci in *Capsella grandiflora*[38].

In addition, a number of experiments supporting Red Queen models have been published over the past 25 years [39-45] and a recent review of these models [46] contains additional references to experimental data. Studies of the New Zealand snail [40], *Potamopyrgus antipodarum* show that mixed populations of sexual and asexual individuals are common in their natural habitat and appear broadly stable. This is compatible with parasite mutation rates at which sexual and clonal populations have the same fitness. An increase in parasite mutation rate would then favour sex, and a decrease would favour clonality. Work on *P*. *antipodarum* has, indeed, suggested that asexual populations prevail in areas associated with low risks of infection (see [46] for references). Two other studies [42,43] have explicitly identified adoption of advantageous mutations as the mechanism for adaptation. Moreover, a recent paper [45] suggests that sex enhances adaptation by allowing independent selection on advantageous and deleterious mutations. This is a major function of sex in the current simulation, with clones suffering adversely when this independent selection is made less efficient. A prediction of the computer simulation is that, where sexual and clonal populations co-exist, the clonal population is significantly polyclonal.

We have looked so far in this Section at biotic environments exemplified by parasites. The computational approach is, however, quite general and can be applied to other biological situations. For example, the sexuality of metazoan parasites can be modelled as a response to the human adaptive immune response [47], which presents NFDS with a high mutation rate. Because of allelic suppression, antibody-producing cells are haploid clones that undergo diversification through somatic hypermutation. The parallel with our computational model appears to be close.

### Final comments

Returning to our original point of departure, namely, the two-fold cost of males identified by Maynard Smith [3], we can see it is only one term in a cost equation. It lacks compensation in static and slowly evolving environments, where clones win, but in dynamic environments that are persistently antagonistic and impose lag loads, the costs of males and outcrossing in sexual reproduction are less than those inflicted by the environment on clonal fitness. The position is made worse for clones as the number of loci increases, and various forms of selective interference are recruited. The comparatively higher fitness of sexual progeny reflects faster adaptation to environmental change, but not for the reasons normally cited. As a result, sex is an Evolutionarily Stable Strategy (ESS) in environments characterized by Red Queens, particularly Red Queens that run fast, but is not an ESS in equilibrium or near-equilibrium environments.

## Conclusions

We develop a computational approach to mutation and selection in a changing environment displaying NFDS. Using this approach, we have been able to identify two complementary parameter spaces that support sex. One space exists where a lag load develops dynamically in a diploid population that cannot fully match an environment mutating endlessly through adoption of novel mutations in a large allele space. The lag load for the clonal population is greater than that for the sexual population, due to a slower rate of adoption of new advantageous mutations. The second parameter space emerges where multiple loci are under independent selection from parasites that impose high mutation rates. This space allows sex to achieve significant comparative advantage over cloning because the polymorphism that develops as a result of the high mutation rate and number of loci causes wastage of advantageous mutations and interference with selection in clonal populations. Both parameter spaces can readily account for situations in which clonal and sexual reproduction are in balance. They also account for the resistance of purely sexual populations to invasion by clones (Maynard Smith’s problem [3]). Both parameter spaces require an environment that has the properties of a Red Queen. The model is compatible with a range of biological data, particularly that stemming from parasites and the evolution of host defences. Because all higher organisms carry parasites, the model has the potential to be completely general.

## Methods

We use a computational approach that employs a genetic algorithm within the class of artificial intelligence (AI) methods known as evolutionary algorithms. A useful summary of the properties, procedural steps, strengths and limitations of genetic algorithms can be found at [32]. Implementation of the genetic algorithm adopts a Wright-Fisher approach using random mating and discrete non-overlapping generations in a single population without geographic effects.

Steps within the implementation are:

a) establish a finite initial population of individuals;

b) evaluate the adaptation or fitness of each individual in that population;

c) apply operators (power function, adaptation/fitness threshold, etc.) where applicable;

d) select best-adapted or fittest individuals stochastically for reproduction (i.e. choose parent(s)), using an adaptation- or fitness-dependent metric;

e) generate haploid gametes for sexual reproduction;

f) breed next-generation individuals by syngamy (sexual reproduction) or mitosis (cloning);

g) apply operators (mutation, crossing-over, etc.) where applicable;

h) repeat steps (b) – (g) until termination (time limit, adaptation steady states, etc.).

The purpose of the genetic algorithm used in this paper is simulation of the behaviour of a host diploid population that optimizes its fitness through adoption of advantageous mutations. The host operates in the presence of a haploid biotic environment that imposes negative frequency-dependent selection (NFDS). This type of biotic environment is typical of those created by parasites and pathogens, where evolution is rapid and antagonistic, but could be extended to organisms that face an adaptive immune system, for example. The biotic environment under investigation is to be distinguished from a physical or non-biotic environment where changes may be acute and severe, but do not have the capacity to evolve. For the sake of brevity, we use the terms “parasite” and “parasite environment” to stand proxy for any biotic environment that has the potential for NFDS.

Hosts, in the simulation, defend themselves from NFDS using resistance alleles. Alleles in host and parasites are conventional number strings (16 × 1-bit), giving 65,536 possible alleles. A diploid host locus encodes symbolically a defence protein with 32 × 1-bit sites (= 2 × 16-bit alleles). Each locus in the host has a corresponding locus in a haploid parasite (possibly best envisaged as an essential parasite protein that enables the parasite to evade host surveillance).

The adaptation of each allele, both host and parasite, (step (b), above) is a numerical score obtained by inverse matching with reference to an opposing set of alleles. For example, a host allele number string, 0110010111011000, has a match score of 10 when measured against a parasite allele number string, 0011000110111010. The parasite allele has the complementary score of 16 – 10 = 6. Thus, adaptation of the alleles of the host species increases as adaptation of alleles representing the parasite declines, and *vice versa*. This is intended to reflect, on a simple numerical scale, the ability of the host to defend itself and the ability of the parasite to evade host defences. Individual allele adaptation scores are calculated against the weighted population of alleles in the counter-species. Both populations are arrested during this reciprocal assessment. Adaptation scores for individual diploid loci are the arithmetic mean of the allele scores; there is no heterozygote advantage. (A locus in this model is not equivalent to that produced by 16 × 1-bit loci because no independent assortment of “1-bit loci” is permitted *within* the 16-bit locus. The two overlap semantically if 16 × 1-bit loci are fully coupled as a haplotype in which crossing-over is forbidden). The simulation described here does not include coupling between the 16-bit alleles to form extended haplotypes that undergo independent assortment. The procedure for deriving adaptation scores for parasite alleles is the same as of that used for hosts.

Adaptation scores are calculated at each generation, for all alleles, and these allele scores are combined, where necessary, to produce scores for individuals. For diploid individuals, the adaptation score is the average of the score for each separate allele. Individuals are chosen stochastically for mating (step (d) above) using a function of their adaptation score. Each individual score can be considered a segment of a linear string, with a length proportional to the adaptation score. Collectively, the sum of all adaptation scores is the overall length of the string. This overall length can be normalized, so that the total length is 1. Individual adaptation scores are reduced *pro rata*. Epistasis can be introduced by subjecting each adaptation score to a power function and re-normalization. Individuals are selected for reproduction using a random number generator between 0 and 1. We use exponents of 1 and 5 for power functions. The higher exponent has the effect of imposing stronger selection on genotypes of lower adaptation. We have not used absolute adaptation or fitness thresholds to truncate populations in these simulations. Stochastic selection creates drift, where low frequency mutations are lost despite intrinsically high adaptation scores.

For sexual reproduction (step (e) above), a diploid parent is chosen randomly on the basis of adaptation score and one allele is chosen at random for transfer to the next generation. This constitutes symbolic formation of a haploid gamete (sperm or egg). A second parent is selected on the same basis and, again, an allele is chosen at random. Parents are retained in their own generation, where they can engage in reproduction on a second or subsequent occasion. Parents cannot breed with themselves. Gender of offspring can be assigned at random to create two sexual populations, but it makes no difference to the computation since the two gender pools are essentially the same in their composition. For clonal reproduction, a selected diploid parent is copied by mitosis. We make a clear distinction between adaptation, which reflects fitness for purpose in both host and parasite, and fitness, which is a multiplicative function of final adaptation score and fecundity. For clones, two individuals are inserted in the next generation for every one inserted by sexual reproduction. This imposes a two-fold fecundity disadvantage on sexual reproduction, equivalent to the two-fold cost of males.

Using the standard language of genetic algorithms [32], mutations are 1-bit alterations in an allele, either 0 to 1 or 1 to 0. The rate at which they occur, *μ*, represents the probability that an allele will undergo a 1-bit alteration per generation. The simulation allows the operator to arrest evolution in the host population while the parasite population undergoes a number of reproductive cycles. This progressively optimizes the adaptation score of the parasite, exposing the host to an abrupt step-jump in parasite pathogenicity when reproduction in the host is restarted. The host effectively suffers imposition of a step lag load that may be substantial. The simulation can also produce random walks in the biotic environment, which has the effect of imposing intermittent lag loads of reversible sign on the host, as opposed to continuous lag loads imposed by NFDS.

Diploid genotypes can also contain multiple loci. Each locus is exposed to independent NFDS imposed by a separate parasite population. These separate populations act to optimize their own adaptation scores and minimize those of the host at each locus. Adaptation scores for each locus are established as for single loci, but adaptation scores for the overall genotype are established multiplicatively. For a fully sexual individual, each locus is uncoupled from all others, to allow independent assortment. In a clonal individual, all loci are coupled. Data available from each run include effective allele number, allele frequency, allele identity, and allele adaptation scores, together with other derived data.

Simulations leading to steady states are run for extended periods of time. (A steady state is one in which the rate of allele mutation and the rate of allele selection have achieved a stable value for some property of the population (adaptation score, allele frequency, etc.). Because mutation and selection are both stochastic, there can be considerable noise in steady-state values. The lengths of time taken to reach and assay the steady state has to be assessed on a case-by-case basis, to avoid biased sampling errors. This is done through sampling over time. Populations with high mutation rates reach steady state rapidly. The longer burn-in times and sampling periods used for slowly mutating populations are usually extended *pro rata* from faster simulations, with a ten-fold decrease in mutation rate resulting in a 10-fold increase in the length of the simulation. The longest simulations have used burn-in periods of >20 million generations and sampling periods of >100 million generations. Simulations are run until the standard errors of the mean become insignificant (numerically less than 0.1% of the highest value). Errors are stated in the figure captions.

The ability of sexual diploid populations to resist invasion by clones was tested by injection of single clones into purely sexual populations, again after a suitable burn-in periods for the sexual starting population. The outcome is described in the legend to Figure 7. In a Wright-Fisher model, the size of the population in each generation is fixed. It starts as 100% sexual, but injection of a clone provides a single individual whose fecundity is progressively offset by an emerging frequency-dependent response that is negative (NFDS). Whether clones win or not reflects the extent to which NFDS acquires sufficient magnitude to shift the adaptation scores of clones to levels at which selection preferentially removes them. A simulation is halted when the whole population is either clonal or sexual. Simulations were repeated for at least 1000 runs. Errors are shown for standard errors of the mean.

The software is written in C. The code is Additional file 1.

### Availability of supporting data

The data set(s) supporting the results of this article is(are) included within the article (and its additional file(s)).

## Competing interests

The authors declare that they have no competing interests.

## Authors’ contributions

DG originated the idea that computer simulation of mutation and selection in an environment displaying negative frequency-dependent selection might give insight into sex as an evolutionary adaptation. CM wrote all the software, and undertook quality checks. DG ran most of the simulations. Technical issues and other issues involving interpretation of the software were discussed jointly. DG wrote the manuscript, which was approved by CM. Both authors read and approved the final manuscript.

## Supplementary Material

Additional file 1 **Software code.** Software codes and assembler to run simulations. Click here for file
